# Efficacy of Mobile Health in Patients With Low Back Pain: Systematic Review and Meta-analysis of Randomized Controlled Trials

**DOI:** 10.2196/26095

**Published:** 2021-06-11

**Authors:** Mingrong Chen, Tingting Wu, Meina Lv, Chunmei Chen, Zongwei Fang, Zhiwei Zeng, Jiafen Qian, Shaojun Jiang, Wenjun Chen, Jinhua Zhang

**Affiliations:** 1 Department of Pharmacy Fujian Medical University Union Hospital Fuzhou China; 2 College of Pharmacy Fujian Medical University Fuzhou China; 3 Department of Neurosurgery Fujian Medical University Union Hospital Fuzhou China

**Keywords:** mobile health, mHealth, low back pain, meta-analysis, pain intensity, disability

## Abstract

**Background:**

Low back pain is one of the most common health problems and a main cause of disability, which imposes a great burden on patients. Mobile health (mHealth) affects many aspects of people’s lives, and it has progressed rapidly, showing promise as an effective intervention for patients with low back pain. However, the efficacy of mHealth interventions for patients with low back pain remains unclear; thus, further exploration is necessary.

**Objective:**

The purpose of this study was to evaluate the efficacy of mHealth interventions in patients with low back pain compared to usual care.

**Methods:**

This was a systematic review and meta-analysis of randomized controlled trials designed according to the PRISMA (Preferred Reporting Items for Systematic Reviews and Meta-analysis) statement standard. We searched for studies published in English before October 2020 in the PubMed, EMBASE, Web of Science, and Cochrane Library databases. Two researchers independently scanned the literature, extracted data, and assessed the methodological quality of the included studies. Bias risks were assessed using the Cochrane Collaboration tool. We used RevMan 5.4 software to perform the meta-analysis.

**Results:**

A total of 9 studies with 792 participants met the inclusion criteria. The simultaneous use of mHealth and usual care showed a better reduction in pain intensity than usual care alone, as measured by the numeric rating scale (mean difference [MD] –0.85, 95% CI –1.29 to –0.40; *P*<.001), and larger efficacy in reducing disability, as measured by the Rolland-Morris Disability Questionnaire (MD –1.54, 95% CI –2.35 to –0.73; *P*<.001). Subgroup analyses showed that compared with usual care, mHealth using telephone calls significantly reduced pain intensity (MD –1.12, 95% CI –1.71 to –0.53; *P*<.001) and disability score (MD –1.68, 95% CI –2.74 to –0.63; *P*<.001). However, without the use of telephone calls, mHealth had no obvious advantage over usual care in improving pain intensity (MD –0.48, 95% CI –1.16 to 0.20; *P*=.16) and the disability score (MD –0.41, 95% CI –1.88 to 1.05; *P*=.58). The group that received a more sensitive feedback intervention showed a significantly reduced disability score (MD –4.30, 95% CI –6.95 to –1.69; *P*=.001).

**Conclusions:**

The use of simultaneous mHealth and usual care interventions has better efficacy than usual care alone in reducing pain intensity and disability in patients with low back pain. Moreover, the results of subgroup analysis revealed that mHealth using telephone calls might play a positive role in improving pain intensity and disability in patients with low back pain.

## Introduction

Low back pain is one of the most common health problems worldwide, and is a main cause of disability according to the latest Global Burden of Disease Study [1]. In 2015, the global prevalence of low back pain with restricted mobility reached 7.3%, indicating that at any given time, low back pain affects 540 million people, representing a wide range of individuals. Low back pain occurs in high-, middle-, and low-income countries among all age groups, ranging from children to older adults [2]. Moreover, in industrialized countries, the lifetime prevalence of nonspecific low back pain is approximately 60%-70%. This not only affects people’s private lives but also causes activity restrictions and work absences. At the same time, low back pain imposes a high economic burden on society [3]. Although physical exercise, conventional therapies, and cognitive behavioral therapy are the most effective nondrug conservative treatments to improve symptoms of low back pain, judging from the implementation of the traditional clinical model of management, the results obtained with these methods are not in line with expectations [4,5]. This finding may be due to the high degree of patient participation required, and the identification of issues and self-management that require patients to follow and complete time-intensive treatment plans independently at home [6]. These components may be the neglected aspects of usual care, which could include occupational therapy; physical therapy; or advice from a general practitioner (eg, family doctor), a specialist, or guidelines.

The term mobile health (mHealth) refers to the use of mobile devices such as mobile phones, patient monitoring equipment, and other wireless devices to provide medical support and health management [7], which may benefit health care providers by exerting positive effects on patient education, diagnosis, and management as components of the health delivery processes [8,9]. The devices for mHealth also include mobile phone software, text messaging, telephone calls, real-time monitoring (eg, motion sensor biofeedback), and network-based game consoles (eg, Nintendo Switch, Nintendo Wii). Compared with the traditional clinical model of management, mHealth can increase the feasibility and rationality of clinical treatment expectations by promoting patients’ adherence to the treatment plan. mHealth can also provide a basis for formulating treatment plans and compensate for the traditional model’s shortcomings to a certain extent [10]. In addition, mHealth can help achieve universal health service coverage by overcoming geographical barriers, thereby increasing the number of paths by which patients can enter medical care systems, and providing medical care services to people in remote areas and communities with insufficient services and inadequate conditions. As no additional medical equipment or time utilization is needed to use mHealth interventions, the expenditures for clinical data monitoring and educational/information exchange between doctors and patients are lower than those of face-to-face services [11]. Moreover, mHealth has been used in many aspects of people’s lives to help them adapt to various health conditions and problems, including those related to mental health [12,13], heart failure [14], and smoking cessation [15,16].

At present, few meta-analyses have been performed on the efficacy of mHealth for patients with low back pain, and therefore the ability to provide more effective or accurate clinical advice is limited. In light of the various advantages of mHealth that are different from usual care and the current global status of low back pain, we performed a meta-analysis to clarify the efficacy of mHealth for patients with low back pain.

## Methods

### Search Strategy

Data were retrieved from the PubMed, Embase, Web of Science, and Cochrane Library databases. We searched for studies in English published until October 2020. The key search strings consisted of two concepts: mHealth and low back pain (Textbox 1). The reference lists of the retrieved studies were checked for identifying further relevant studies.

**Example of the search strategy (EMBASE).**

- Search 1: (“mobile application” OR “telemedicine” OR “text messaging” OR “mobile phone” OR “smartphone” OR “social media” OR “internet”)/exp
- Search 2: (mobile OR “portable software application” OR tele* OR mhealth OR ehealth OR “e health” OR “mhealth” OR ?phone* OR text* OR “short message” OR sms OR app OR apps OR digital* OR web* OR internet* OR ?media OR wireless OR computer OR video* OR bluetooth OR blog* OR online OR electronic OR “mp3 player” OR “mp4 player” OR wechat OR whatsapp OR twitter OR “virtual reality” OR “interactive voice response” OR facebook OR networking): title/abstract/keywords
- Search 3: Search 1 OR Search 2
- Search 4: “low back pain”/exp OR “backache”/exp
- Search 5: (“low back pain” OR “low back ache” OR “low backache” OR “lower back pain” OR “back disorder” OR backache OR “back pain” OR lumbago OR dorsalgia OR coccyx OR coccydynia OR sciatica OR ischialgia OR spondylosis): title/abstract/keywords
- Search 6: Search 4 OR Search 5
- Search 7: Search 3 AND Search 6

### Study Inclusion Criteria

The following inclusion criteria were used: (1) the study design was a randomized controlled trial (RCT); (2) mHealth (eg, mobile phone, computer, motion sensor biofeedback, and network-based game consoles) and usual care (eg, exercise and/or advice) were used simultaneously in the experimental group, and usual care or usual care and placebo were used in the control group; (3) participants were confirmed to have low back pain; and (4) the outcomes were measured using the numeric rating scale (NRS) and/or Roland-Morris Disability Questionnaire (RMDQ), with data expressed as mean (SD). Two researchers selected the studies independently in accordance with the above criteria.

### Study Exclusion Criteria

Studies were excluded from the meta-analysis if: (1) not all of the participants were diagnosed with low back pain; (2) the study population included pregnant women or patients recovering after spinal surgery; or (3) email had been used as an intervention for office workers in the workplace. The latter criterion was based on previous studies [17-20] indicating a consistently high degree of patient compliance. We believe that the specific combination of being an office worker and receiving email at the workplace conveys a highly sensitive nature of the feedback, resulting in high patient compliance. This finding is inconsistent with the general usage of email in mHealth and is not universal. Therefore, this meta-analysis did not include interventions for office workers using email in the workplace.

### Data Extraction

The required data were extracted independently by two researchers and crosschecked to avoid potential data extraction errors. Disagreements during the extraction process were resolved by seeking the opinion of a third researcher. The extracted information included the first author’s name, year of publication, sample size, age, sex ratio, and participants’ scores on the NRS and RMDQ. The final postintervention data with the longest follow-up time was used in our analysis if the study (ie, [21-23]) reported results from a different period.

### Data Analysis

We assigned participants who used telephone calls, internet/email, mobile phones, or other mHealth methods and usual care at the same time to the mHealth experimental group, and those who used usual care alone to the control group. RevMan 5.4 software was used for the meta-analysis, with the mean difference (MD), SD, and 95% CI as the statistics of interest. The overall pooled effect estimate was assessed using *Z*‐statistics, and statistical significance was considered at *P*<.05. The χ^2^ test was used to examine the heterogeneity of the results: if *P*≥.10 and I^2^≤50%, a fixed-effects model was used for the meta-analysis; otherwise, a random-effects model was used.

Given reports of positive effects of telephone calls on patients’ self-management and compliance [24], we defined subgroups based on two indicators: whether the intervention used telephone calls or more sensitive feedback methods (ie, motion sensor biofeedback). We examined the effect of telephone calls on the following outcomes: (1) whether the use of telephone calls affects NRS scores (the experimental group using mHealth was divided into two groups based on use of telephone calls and no telephone calls); and (2) whether the use of telephone calls or more sensitive feedback interventions affects RMDQ scores (the experimental group using mHealth was divided into three groups based on use of telephone calls, no telephone calls, and use of more sensitive feedback interventions such as motion sensor biofeedback).

### Quality Assessment

The risk of bias was assessed using the Cochrane Collaboration Tool for Assessing Risk of Bias in Randomised Trials [25]. We evaluated seven aspects of the studies: random sequence generation, allocation concealment, blinding of participants and personnel, blinding of outcome assessments, incomplete outcome data, selective reporting, and other biases. Risk of bias ratings of “low,” “high,” and “unclear” were assigned to each aspect of each study. Two researchers performed the quality assessments of the included studies. Differences in opinions of the two researchers were resolved through discussion and decision. A third researcher was consulted for disagreements, and a decision was made after a discussion.

## Results

### Study Selection

**Figure 1:**
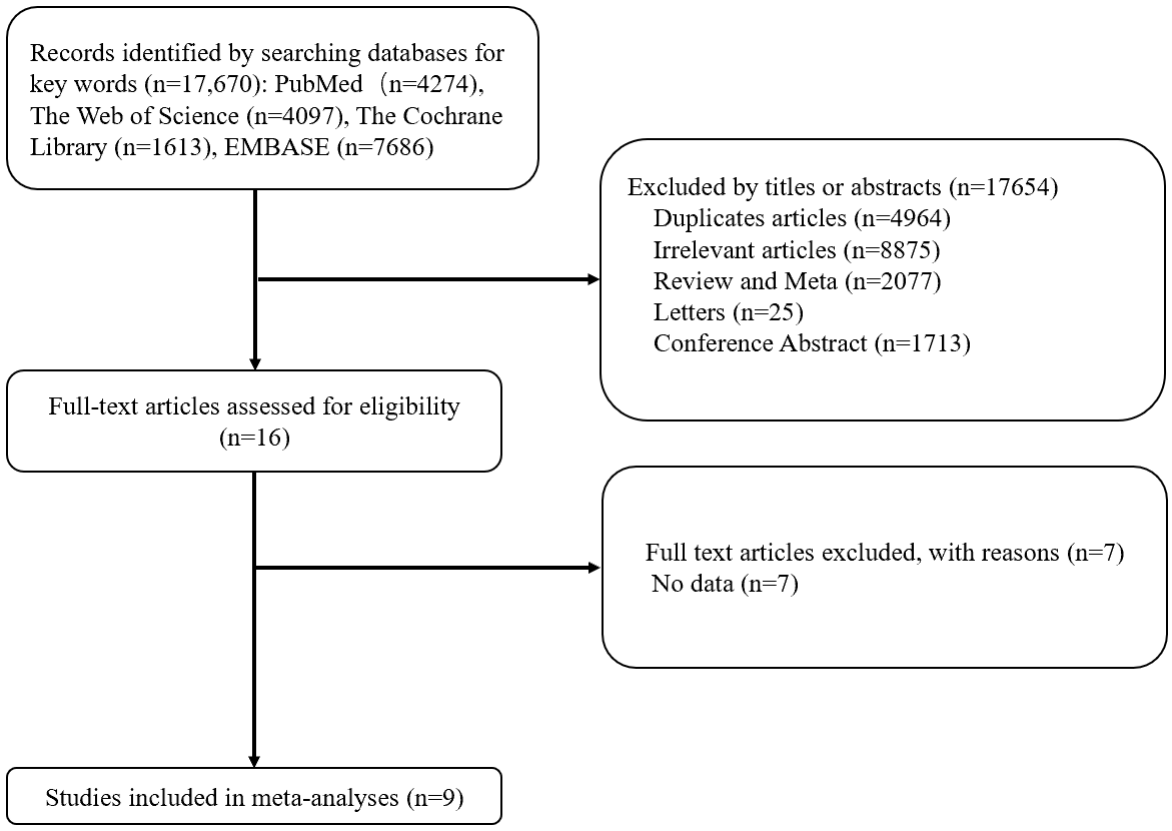
Flowchart of the screening and selection of studies.

A total of 17,670 studies were identified during the initial examination. After all studies were screened and filtered using the inclusion and exclusion criteria, 9 studies were included in the final meta-analysis. These studies comprised 792 participants, 407 of whom were allocated to the mHealth group and 385 were allocated to the usual care group, as shown in the flowchart in Figure 1.

### Study Characteristics

Features of the included studies and outcome data are summarized in Multimedia Appendix 1 and Table 1, respectively. Of the 9 included studies, 3 from research institutions were performed in Australia [21,22,26]; 1 was performed in the United States [23]; and 1 study each was performed in India, China, Brazil, the United Kingdom, and Italy. The proportion of female participants ranged from 50% to 74%, and the mean age of the participants ranged from 40 to 68 years. The follow-up period of the included studies ranged from 4 weeks to 12 months.

**Table 1:** Pain and disability scores at baseline and after the interventions.

Study	Pain intensity: NRS^a^ (score range 0-10), mean (SD)					Disability: RMDQ^b^ (score range 0-24), mean (SD)				
	mHealth^c^ group		Control group		mHealth group				Control group	
	Baseline	After intervention	Baseline	After intervention	Baseline		After intervention	Baseline		After intervention
Amorim et al [26]	5.3 (1.9)	3.8 (2.4)	5.1 (1.4)	4.0 (3.4)	8.9 (5.4)		5.7 (5.3)	9.0 (6.1)		6.0 (5.7)
Bernardelli et al [27]	NA^d^	NA	NA	NA	6.3 (4.4)		3.8 (3.9)	6.4 (4.9)		4.3 (4.2)
Chhabra et al [28]	7.3 (1.9	3.3 (1.7)	6.6 (2.1)	3.2 (2.7)	NA		NA	NA		NA
Damush et al [23]	NA	NA	NA	NA	14.7 (6.7)		9.1 (6.8)	13.9 (6.8)		11.3 (8.1)
Geraghty et al [29]	Ⅰ: 4.0 (2.6); Ⅱ:4.5 (2.6)	Ⅰ: 3.6 (2.5); Ⅱ: 3.1 (2.3)	3.6 (3.1)	4.0 (2.5)	Ⅰ: 6.6 (4.6); Ⅱ: 7.7 (4.7)		Ⅰ: 5.8 (4.5); Ⅱ: 5.1 (5.1)	6.8 (4.9)		6.3 (5.1)
Kent et al [21]	NA	NA	NA	NA	11.8 (8.8)		7.2 (2.6)	11.3 (7.0)		11.0 (1.3)
Monteiro-Junior et al [30]	6.5 (1.1)	1.7 (1.9)	6.6 (1.2)	1.4 (2.9)	NA		NA	NA		NA
Petrozzi et al [22]	5.1 (1.8)	3.0 (2.1)	4.9 (2.0)	4.0 (2.1)	9.9 (4.2)		4.2 (3.7)	9.9 (4.7)		5.3 (5.1)
Yang et al [31]	NA	NA	NA	NA	6.00 (3.74)		4.40 (3.05)	12.00 (3.61)		11.70 (5.69)

^a^NRS: numeric rating scale.

^b^RMDQ: Rolland-Morris Disability Questionnaire.

^c^ mHealth: mobile health.

^d^NA: not applicable (not assessed).

### Risk of Bias in the Included Studies

The Cochrane Collaboration Risk of Bias Tool was used to assess the risk of bias of the 9 included studies. Seven studies used computer-generated random numbers [21,22,26-30] and 2 studies did not indicate use of a sequence generation method [23,31]. Two studies did not report allocation concealment [23,30]; therefore, the risk of selection bias was relatively low. The risk of performance bias was found to be relatively high. Participants were blinded to the treatment conditions in 4 of the studies [21,23,29,30], 2 studies lacked sufficient information about whether the participants were blinded [26,27], and participants were not blinded in 3 studies [22,28,31]. Investigators were blinded to the outcomes in all of the included studies. An unclear risk of attrition bias was found in 1 study [31] and a high risk was found in 2 studies, as the dropout rate was relatively high [26,29]. The risk for reporting bias was low in all studies, and the risk for other types of bias was high in 1 study [28].

**Figure 2:**
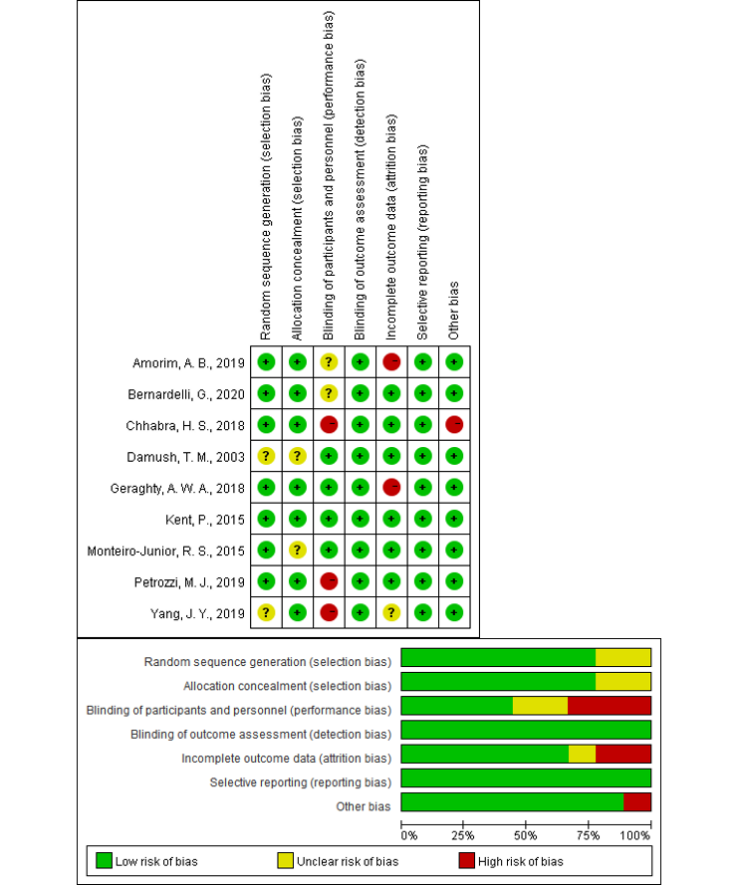
Risk assessment of bias in the included studies.

The overall risk of bias was relatively low, but performance bias was relatively high, as 3 of the studies used a single-blinded method [22,28,31] (Figure 2). However, it should be noted that the effects of blinding on the results of studies in the fields of rehabilitation and physical therapy are currently unclear [32,33].

### Meta-analysis

#### Comparison of Pain Intensity

**Figure 3:**
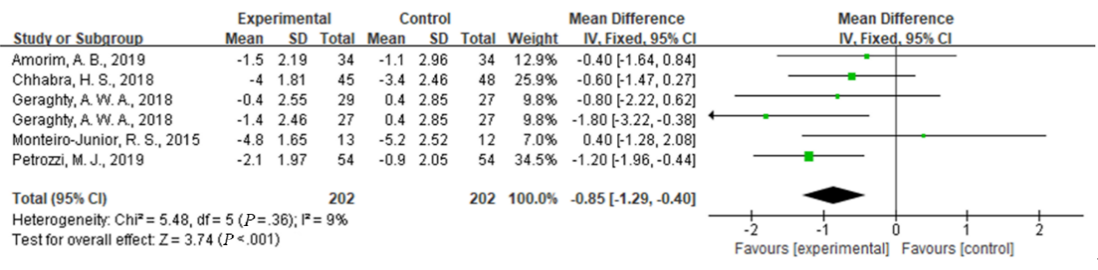
Forest plot of the efficacy of mobile health and traditional health interventions in reducing pain intensity.

Compared with usual care, the simultaneous interventions of mHealth and usual care were more effective in reducing pain, as indicated by the NRS scores of 404 participants in 5 studies (MD –0.85, 95% CI –1.29 to –0.40; I^2^=9%; *P*<.001) (Figure 3).

#### Comparison of Disability

**Figure 4:**
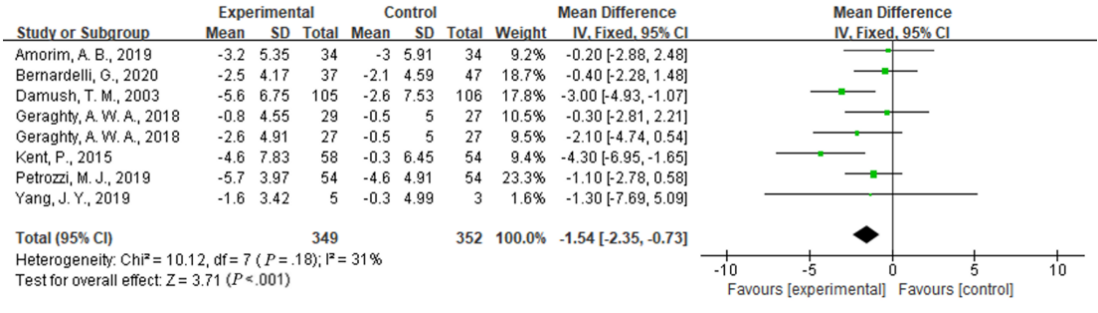
Forest plot of the efficacy of mobile health and traditional health interventions on disability.

Compared with usual care, the simultaneous interventions of mHealth and usual care had a larger effect on reducing disability in patients with low back pain, as indicated by the RMDQ scores of 885 participants in 8 studies (MD –1.54, 95% CI –2.35 to –0.73; I^2^ =31%; *P*<.001) (Figure 4).

#### Subgroup Analysis

**Figure 5:**
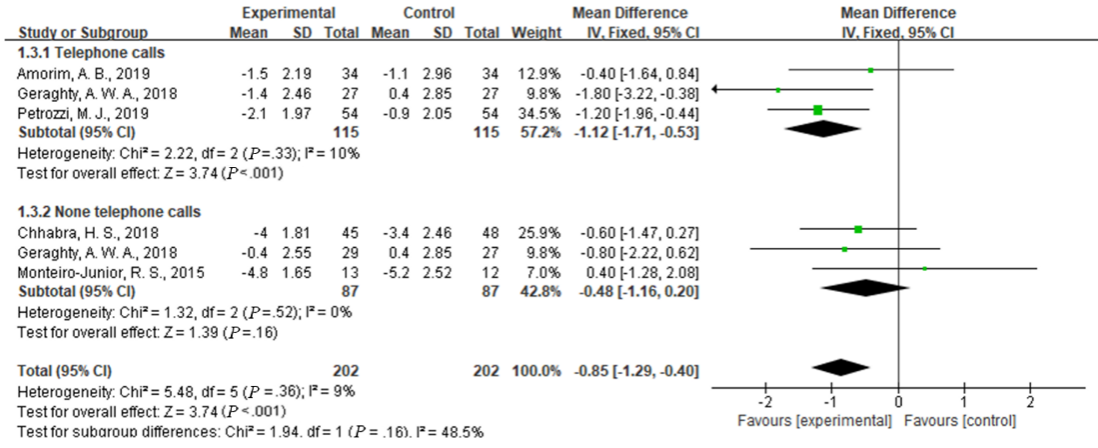
Efficacy of telephone calls use in reducing pain intensity.

We evaluated pain intensity, as measured by the NRS, to examine the efficacy of mHealth using telephone calls. We performed a subgroup analysis of participants in the mHealth experimental group that used telephone calls and those who were in an intervention group that did not use telephone calls. A difference was found between the telephone group and nontelephone group, although it was not statistically significant (I^2^=48.5%, *P*=.16). Compared with usual care, mHealth using telephone calls significantly reduced pain intensity in 3 studies (MD –1.12, 95% CI –1.71 to –0.53; I^2^ =10%, *P*<.001); however, without the use of telephone calls, mHealth had no obvious advantage over usual care (MD –0.48, 95% CI –1.16 to 0.20; I^2^ 0%, *P*=.16) (Figure 5).

**Figure 6:**
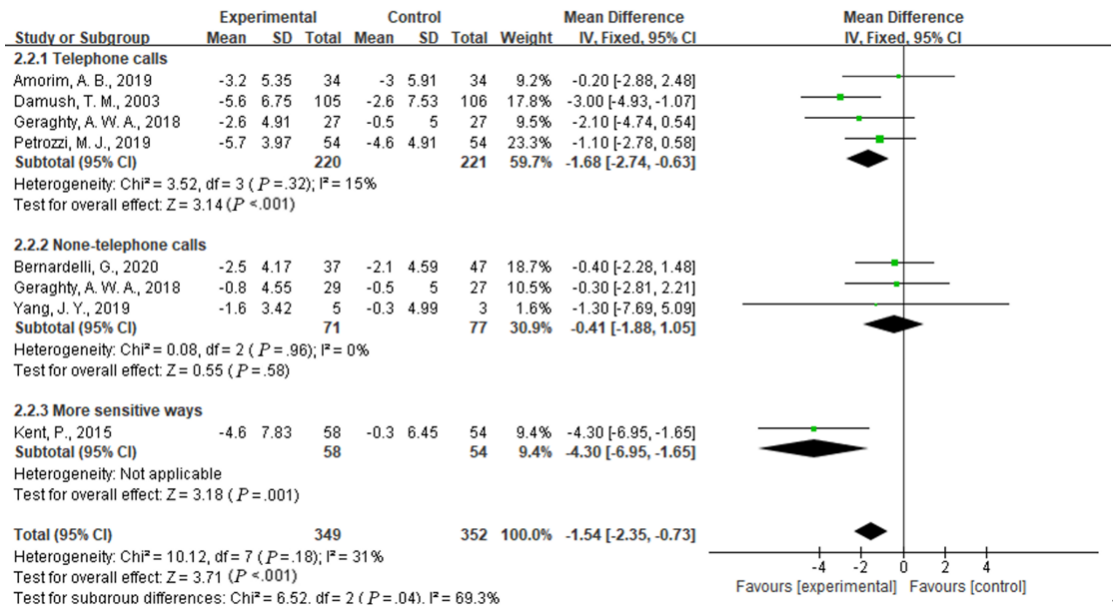
Efficacy of telephone use and the more sensitive feedback intervention in reducing disability.

We evaluated disability, as measured by the RMDQ, to examine the efficacy of different types of mHealth. We performed a subgroup analysis of participants in the mHealth experimental group that used telephone calls, did not use telephone calls, or used a more sensitive feedback intervention. The analysis indicated a significant difference between the telephone calls group, the more sensitive feedback intervention group, and the nontelephone group (I^2^=69.3%, *P*=.04). Compared with the group that received usual care, the experimental mHealth group that involved telephone calls showed a significantly reduced disability score in 4 studies (MD –1.68, 95% CI –2.74 to –0.63; I^2^ = 15%, *P*<.001). The group that received a more sensitive feedback intervention showed a significantly reduced disability score in 1 study (MD –4.30, 95% CI –6.95 to –1.69; *P*=.001), and the group that did not receive an intervention with telephone calls showed no significant difference in their RMDQ scores from the two other groups in 3 studies (MD –0.41, 95% CI –1.88 to 1.05; I^2^=0%, *P*=.58) (Figure 6).

#### Publication Bias and Sensitivity Analysis

A sensitivity analysis was performed using one-by-one elimination of studies that reported the outcomes of the NRS and the RMDQ. No significant change was found in the outcomes, indicating that the results were stable. According to the Cochrane Group, a funnel plot to detect publication bias is not recommended when fewer than 9 studies are included in a meta-analysis [34]. Hence, a funnel plot was not used to detect publication bias because of the small number of studies in our meta-analysis.

## Discussion

### Principal Findings

This meta-analysis of 9 studies with 792 patients revealed a significant positive effect of the simultaneous interventions of mHealth and usual care compared with usual care only on patients with low back pain. The mHealth intervention performed significantly better than usual care on measures of pain intensity (MD –0.85, 95% CI –1.29 to –0.40; I^2^=9%, *P*<.001) and disability (MD –1.54, 95% CI –2.35 to –0.73; I^2^=31%, *P*<.001). The subgroup analysis of the scores on the NRS and RMDQ showed that the use of telephone calls or more sensitive feedback devices for the intervention might be superior to other types of mHealth interventions or usual care in terms of improving pain intensity and disability. This conclusion is consistent with a study by Niznik et al [24] who reported advantages of telephone calls, and concluded that telephone calls have positive effects on clinical disease management, patient management, and patient compliance. This study has similarities and differences with the results of another study [35] that examined differences in mHealth between a web-based health program and an mHealth-based program. No significant effects on pain or disability were found among participants in the web health program versus the controls. Compared with the controls, the participants in the trials on mHealth-based programs reported clinically significant effects on pain intensity and disability.

Our study demonstrates the importance of telephone calls in mHealth. We believe that telephone calls may be one of the main effective types of mHealth with great positive effects on patients in reducing pain and disability. As one of the mobile medical methods, telephone calls might be superior to other types of mHealth for the following reasons. First, according to Simblett et al [36], a major challenge of mHealth is the high dropout rate of participants with sensors and the usability of apps. Therefore, in contrast to other types of mHealth, we believe that active telephone calling from the treatment unit instead of the passive use of software and websites can greatly improve the enthusiasm and compliance of patients, thereby ensuring a positive impact of mHealth on patients. Another option is to use motion sensor biofeedback to achieve real-time communication and feedback to improve efficacy, which is consistent with the opinion of Sim [37]. Second, mHealth has potential to facilitate the achievement of universal coverage for health services by overcoming geographical barriers, increasing the number of pathways to medical care, and providing medical care services to people in remote areas and communities with insufficient services and inadequate conditions [11]. However, in most countries, especially in remote areas, the network infrastructure is far less stable than telephone calls. The costs in money and time of developing software or websites in business or medical settings are much higher than those associated with telephone calls.

To our knowledge, subgroup analyses of the efficacy of telephone calls have not been performed to date. Since few studies have examined the impact of mHealth on patients with low back pain, there are no related articles for comparison. Only a systematic review and meta-analysis of 5 articles performed by Du et al [35] on the effectiveness of mHealth in the self-management of patients with chronic low back pain was found in the published literature. The results revealed that mHealth-based self-management might play a positive role in improving short-term pain intensity and short-term disability in patients with chronic low back pain. After careful reading of this meta-analysis, we found similarities and differences with our study. The endpoint data of the included RCTs were also extracted by Du et al [35] for the meta-analysis. However, in contrast to their study, we included more articles in our meta-analysis, and calculated differences between the baseline and endpoint data of each included RCT. We believe that this method is more accurate and that it further supports our conclusion. Most previous meta-analyses related to mHealth did not distinguish between the use of mHealth alone and the simultaneous use of mHealth and routine care, nor did they restrict the intervention methods of the control group. We chose usual care and mHealth for the intervention group, and usual care only for the control group, and we believe that such a comparison yielded conclusions that are more reliable than other comparisons.

Establishing clinical relevance is the key to whether mHealth can be used in patients with low back pain. Yet, small effects (–0.85) are observed at the group level for pain intensity when compared to the control group, which do not meet the minimal clinically important difference criterion of –1.77 [38]. However, as one of the intervention methods used simultaneously with usual care, mHealth can significantly improve the curative effect in reducing pain intensity and disability in patients with low back pain, while reducing human resources and time costs. Therefore, this method is worthy of adoption.

The objective of this study was to examine the influence of mHealth interventions on the pain intensity and disability of patients with low back pain. Our investigation highlights differences between the intervention of usual care alone and the simultaneous use of usual care and mHealth. Compared with using usual care alone, the intervention of telephone calls had a significant beneficial effect on patients’ disability. These findings are expected to provide guidance for clinical decisions and contribute to this field.

### Limitations

Our study has several limitations. First, this meta-analysis may be biased if the literature search failed to identify all trials reporting on differences between mHealth and usual care or if the selection criteria for including trials were applied in a subjective manner. To reduce these risks, we performed thorough searches across multiple literature databases and clinical trial databases, and used explicit criteria for study selection and data extraction and analysis. Second, mHealth may have specific effects that vary by the type of low back pain. That is, to better evaluate the efficacy, and save human resources and time costs, passive sensing in mHealth may be more suitable for chronic low back pain, whereas active sensing may be more suitable for acute low back pain, which can be administered multiple times a day to capture short-term variations in responses [37]. However, owing to the insufficient number of studies on acute and subacute low back pain, we were unable to perform a subgroup analysis according to the type of back pain, and therefore this issue should be examined in the future. Finally, as this was a study-level rather than participant-level meta-analysis, we were able to analyze univariate associations, but not multivariate associations of baseline features with outcomes.

### Conclusion

The results of this meta-analysis suggest that the simultaneous interventions of mHealth and usual care, compared with usual care alone, are significantly better for reducing pain intensity and disability in patients with low back pain. The use of telephone calls or more sensitive feedback interventions may further increase the positive effects of these simultaneous interventions on the disability of patients with low back pain. The wider use of mHealth may contribute significantly to the population of patients with low back pain. Therefore, the simultaneous interventions of mHealth and usual care may be a promising method worth considering.

## Appendix

Multimedia Appendix 1 Characteristics of the included studies.

## Abbreviations

MD: mean difference
mHealth: mobile health
NRS: numeric rating scale
RCT: randomized controlled trial
RMDS: Roland-Morris Disability Questionnaire
