# Supplementary material for: Efficacy of Mobile Health in Patients With Low Back Pain: Systematic Review and Meta-analysis of Randomized Controlled Trials
Source: JMIR Mhealth Uhealth. 2021 Jun 11;9(6):e26095. doi: 10.2196/26095 (PMC8235295; doi:10.2196/26095)
Supplement: Multimedia Appendix 1 [file mhealth_v9i6e26095_app1.docx]

Table 1. Characteristics of the included studies.

| **Study** | **Sample Size** | | **Age (mean or median), years** | | **Female (%)** | | Intervention modes | Intervention frequency | Follow-up duration |
| --- | --- | --- | --- | --- | --- | --- | --- | --- | --- |
|  | ^a^ mHealth group | ^b^Control group | mHealth group | Control group | mHealth group | Control group |  |  |  |
| Amorim  (2019)[26] | 34 | 34 | 59.5 (11.9) | 57.1 (14.9) | 44 | 56 | Phone application and telephone  **Duration:6 months** | Fortnightly | 6 months |
| Bernardelli  (2020)[27] | 37 | 47 | 51.9 (8.1) | 50.5 (9.7) | 78 | 72 | A video on the company’s website  **Duration: 7 weeks** | Weekly | 7 weeks |
| Chhabra  (2018)[28] | 45 | 48 | 41.4 (14.2) | 41.0 (14.2) | NA | | Smartphone app  **Duration: 12 weeks** | Daily | 12 weeks |
| Damush  (2003)[23] | 105 | 106 | 45.4 | 45.5 | 71.7 | 75.2 | Telephone follow-up  **Duration: 11 months** | at 4, 6, 8 week, after which monthly | 12 months |
| Geraghty  (2018)[29] | 29/27 | 27 | 54.5/59.3 | 60.3 | 65.2/63.0 | 55.6 | Software on PC and telephone  **Duration: 6 weeks** | per week for software, three calls | 3 months |
| Kent  (2015)[21] | 58 | 54 | 39 (12) | 48 (12) | 52 | 57 | Motion-sensor biofeedback and software application  **Duration: 10 weeks** | Weekly | 52 weeks |
| Monteiro-Junior  (2015)[30] | 13 | 12 | 68 | | NA | | Wii - based exercise  **Duration: 8 weeks** | 3 times a week | 8 weeks |
| Petrozzi  (2019)[22] | 54 | 54 | 50.1 (12.8) | 50.6 (14.4) | 53.7 | 59.3 | Website and telephone  **Duration: 8 weeks** | Weekly | 12 months |
|  |  |  |  |  |  |  |  |  |  |
|  |  |  |  |  |  |  |  |  |  |
|  |  |  |  |  |  |  |  |  |  |
| Yang  (2019)[31] | 5 | 3 | 35.00 (10.93) | 50.33 (9.29) | 20 | 100 | Smartphone based app  **Duration: 4 weeks** | 4 times per day | 4 weeks |
